# Targeting the *BRCA1*/*2* deficient cancer with PARP inhibitors: Clinical outcomes and mechanistic insights

**DOI:** 10.3389/fcell.2023.1133472

**Published:** 2023-03-22

**Authors:** Ashwin Ragupathi, Manrose Singh, Alexis M. Perez, Dong Zhang

**Affiliations:** Department of Biomedical Sciences, College of Osteopathic Medicine, New York Institute of Technology, Old Westbury, NY, United States

**Keywords:** BRCA1 and BRCA2, PARP1, PARP inhibitors, synthetic lethality, breast cancer, ovarian cancer, pancreatic cancer, prostate cancer

## Abstract

BRCA1 and BRCA2 play a critical role in a variety of molecular processes related to DNA metabolism, including homologous recombination and mediating the replication stress response. Individuals with mutations in the *BRCA1* and *BRCA2* (*BRCA1/2*) genes have a significantly higher risk of developing various types of cancers, especially cancers of the breast, ovary, pancreas, and prostate. Currently, the Food and Drug Administration (FDA) has approved four PARP inhibitors (PARPi) to treat cancers with *BRCA1/2* mutations. In this review, we will first summarize the clinical outcomes of the four FDA-approved PARPi in treating *BRCA1/2* deficient cancers. We will then discuss evidence supporting the hypothesis that the cytotoxic effect of PARPi is likely due to inducing excessive replication stress at the difficult-to-replicate (DTR) genomic regions in *BRCA1/2* mutated tumors. Finally, we will discuss the ongoing preclinical and clinical studies on how to combine the PARPi with immuno-oncology drugs to further improve clinical outcomes.

## 1 Introduction

In December of 2014, the Food and Drug Administration (FDA) approved olaparib (LYNPARZA, KuDOS/AstraZeneca), a first-in-class PARP inhibitor (PARPi), to treat patients with advanced ovarian cancers and also with *BRCA1/2* mutations who have previously been treated with multiple rounds of chemotherapy (Kim et al., 2015). This marked a tremendous milestone for cancer therapy for several reasons. (1) It is the first-ever personalized and targeted therapy for treating ovarian cancers. (2) It is also the first precision oncology drug that exploits targets in DNA damage response (DDR) and DNA repair pathways. (3) It validates the foresighted concept of synthetic lethality in oncology drug development that Friend and colleagues first articulated in the late 1990s (Hartwell et al., 1997). In this review, we first summarize and update the clinical trial results of the four FDA approved PARPi in treating cancers with *BRCA1* and *BRCA2 (BRCA1/2)* mutations, including cancers of the breast, ovary, pancreas, and prostate. We then discuss the hypothesis that PARPi exerts its cytotoxic effect by instigating excessive replication stress at the difficult-to-replicate (DTR) genomic regions in the *BRCA1/2* mutated tumors, eventually leading to cell death. Finally, we briefly discuss furthering the potential of PARPi in the context of immune-oncology.

## 2 Clinical outcomes of the four FDA approved PARP inhibitors in treating the *BRCA1/2* mutant cancers

Individuals with germline mutations in the *BRCA1*/*2* genes are at a higher risk in developing various types of cancers, including cancers of the breast, ovary, pancreas, and prostate (Couch et al., 2014; King, 2014). The FDA has approved four PARPi to treat tumors with *BRCA1/2* mutations, including olaparib, rucaparib, niraparib, and talazoparib (Table 1). In this section, we summarize and update the results of Phase III clinical trials of the four FDA-approved PARPi in treating *BRCA1/2* deficient cancers (Table 2). We also briefly describe FDA-approved companion tests for eligibility in these phase III clinical trials. BRCA mutations are also present in numerous other cancers such as lung, endothelial, and Acute myeloid leukemia (reviewed in PMID: 33015058), but were not reviewed due to a lack of FDA-approved PARP inhibitors for therapy. For a comprehensive list of clinical trials related to the PARPi, please visit clinicaltrials.gov.

**TABLE 1:** The four FDA approved PARP inhibitors.

			Mechanism of action	
	Brand name	Manufacture	Catalytic inhibition (PARP1)^*^	Trapping^*^
Niraparib	ZEJULA	Merck/Tesaro/GSK	Yes (+)	Yes (3X)
Olaparib	LYNPARZA	KuDOS Pharmaceutical/AstraZeneca	Yes (++)	Yes (1X)
Rucaparib	RUBRACA	Agouron Pharmaceuticals/Clovis Oncology	Yes (+++)	Yes (1X)
Talazoparib	TALENNA	LEAD Therapeutics/Pfizer	Yes (++++)	Yes (100X)

Notes: “+” signs indicate the qualitative potency of inhibiting the enzymatic activity of PARP1. “1X, 3X, and 100X” indicates the relative potency of trapping PARP1 on DNA.

*Rudolph, J.; Jung, K.; Luger, K., Inhibitors of PARP: Number crunching and structure gazing. *Proc Natl Acad Sci U S A* **2022,** *119* (11), e2121979119.

**TABLE 2:** Summary of the Phase III clinical trials for the four *BRCA1/2* mutated cancers (ovarian, breast, prostate, and pancreatic).

Clinical Trial	Drug	Indication	Treatment Arm	Total Patients [n]	Treatment Group [n]	Treatment Group Mutations [n/%]	Setting	Results	Citation
Ovarian Cancer									
SOLO2/ENGOT-Ov21 NCT01874353	Olaparib	Maintenance	Olaparib 300 mg BID vs. Placebo	295	196	BRCA1: 132 (67%) **BRCA2**: 58 (30%) **MI**: 6 (3%)	Platinum-sensitive, relapsed ovarian cancer (HGSOC or HGEOC) with BRCA1/2 mutation; ≥ **2** Platinum therapy regimens	**Median PFS** 19.1 vs. 5.5 months (*p* < 0.0001)	Pujade-Lauraine et al. (2017)
SOLO1/GOG 3004 NCT01844986	Olaparib	Maintenance	Olaparib 300 mg BID vs. Placebo	391	260	**BRCA1**: 191 (73%) **BRCA2**: 66 (25%) Both: 3 (1%)	Platinum-sensitive, relapsed ovarian cancer (HGSOC or HGEOC) with BRCA1/2 mutation; 1 Platinum therapy regimens	**Median PFS** 56.0 vs. 13.6 months (*p* < 0.001)	Moore et al. (2018), DiSilvestro et al. (2022)
ENGOT-OV16/NOVA NCT01847274 (gBRCA Cohort)	Niraparib	Maintenance	Niraparib 300 mg Daily vs. Placebo	203	138	**BRCA1**: 85 (61.6%) **BRCA2**: 51 (37.0%) **BRCA1/2 Rearrangement**: 9 (6.5%)	Platinum-sensitive recurrent HGSOC; primary peritoneal cancer, or fallopian-tube cancer	**Median PFS** 21.0 vs. 5.5 months (*p* < 0.001)	Mirza et al. (2016)
ARIEL3 NCT01968213 (BRCA-Mutant cohort)	Rucaparib	Maintenance	Rucaparib 600 mg BID vs. Placebo	196	130	**BRCA1**: 80 (61.5%) **BRCA2**: 50 (38.0%)	Platinum-sensitive recurrent HGSOC or HGEOC, primary peritoneal or fallopian tube cancer	**Median PFS** 16.6 vs. 5.4 months (*p* < 0.0001)	Coleman et al. (2017)
ARIEL4 NCT02855944 (Efficacy Population)	Rucaparib	Monotherapy	Rucaparib 600 mg BID vs. Chemotherapy	325	220	**BRCA1**: 173 (79%) **BRCA2**: 47 (21%)	High-grade epithelial ovarian, fallopian tube, or primary peritoneal cancer	**Median PFS** 7.4 vs. 5.7 months (*p* < 0.001)	Kristeleit et al. (2022)
Breast Cancer									
OlympiAD NCT02000622	Olaparib	Monotherapy	Olaparib 300 mg BID vs. Standard Therapy	302	205	**BRCA1**: 117 (57.1%) **BRCA2**: 84 (41%) **Both**: 4 (2.0%)	HER2-negative metastatic breast cancer with gBRCA1/2 mutations	**Median PFS** 7.0 vs. 4.2 months (*p* < 0.001)	Robson et al. (2017)
OlympiA NCT02032823	Olaparib	Maintenance	Olaparib 300 mg BID vs. Placebo	1836	921	**BRCA1**: 657 (71.3%) **BRCA2**: 261 (28.3%) **Both**: 2 (0.2%)	HER2-negative early breast cancer with gBRCA1/2, received prior local treatment and neoadjuvant or adjuvant chemotherapy	3-year IDFS 85.9% vs. 77.1% (*p* < 0.001) **3-year DDFS** 87.5% vs. 80.4% (*p* < 0.001) **4-year OS** 89.8% vs. 86.4% (*p* = 0.009)	Tutt et al. (2021), Geyer et al. (2022)
EMBRACA NCT01945775	Talazoparib	Monotherapy	Talazoparib 1 mg Daily vs. Standard Therapy	431	287	**BRCA1**: 133 (46.3%) **BRCA2**: 154 (53.7%)	HER2-negative metastatic breast cancer with gBRCA1/2 mutations	**Median PFS** 7.0 vs. 4.2 months (*p* < 0.001) **ORR** 62.6% vs. 27.2% (*p* < 0.001)	Litton et al. (2018)
**Prostate Cancer**									
PROfound NCT02987543 (Cohort A)	Olaparib	Monotherapy	Olaparib 300 mg BID vs. Standard Therapy	245	162	**BRCA1**: 8 (5%) **BRCA2**: 80 (49%) **ATM**: 66 (37%)	mCRPC with disease progression while receiving a new enzalutamide or abiraterone treatment	**Imaging-based Median PFS** 7.4 vs. 3.6 months (<0.001) **Median OS** 18.5 vs. 15.1 months (*p* = 0.02)	de Bono et al. (2020)
**Pancreatic Cancer**									
POLO NCT02184195	Olaparib	Maintenance	Olaparib 300 mg BID vs. Placebo	154	92	**BRCA1**: 29 (32%) **BRCA2**: 62 (67%) **Both**: 1 (1%)	BRCA1/2 mutations and metastatic pancreatic cancer and disease that had not progressed during first-line platinum-based chemotherapy	**Median PFS** 7.4 months vs. 3.8 months (*p* = 0.004)	Golan et al. (2019)

Abbreviations = BID: bid in die (or twice daily); PFS: Progression free Survival; OS: Overall Survival; IDFS: Invasive Disease-Free Survival; DDFS: Distant Disease-Free Survival; HGSOC: High Grade Serous Ovarian Cancer; HGEOC: high-grade endometrioid cancer; mCRPC: Metastatic castration-resistant prostate cancer; gBRCA: germline BRCA mutated; MI: Missing Information

### 2.1 Ovarian cancer

Ovarian cancer accounts for 2.5% of all malignancies among women but accounts for 5% of total female cancer deaths (Torre et al., 2018). Due to general and non-specific symptoms of the early disease and the lack of early detection screening options, patients usually present with advanced disease at the time of diagnosis. Approximately 18% of epithelial ovarian cancer cases, particularly high-grade serous carcinomas, are due to inherited mutations in *BRCA1*/*2* genes; these mutations account for almost 40% of ovarian cancer cases in women with a family history of the disease (Couch et al., 2014). Currently, the FDA has approved olaparib, niraparib, and rucaparib for treating and managing *BRCA*-mutated ovarian cancers. In December 2014, the FDA approved the first PARP inhibitor, olaparib, as a monotherapy for patients with advanced *BRCA1/2*-mutated ovarian cancer who were treated with three or more prior lines of chemotherapy based on the promising results from a Phase II clinical trial (NCT0107662) (Kaufman et al., 2015; Arora et al., 2021). The FDA then expedited the approval of rucaparib and niraparib based on the ARIEL2 and Study 10 clinical trials for rucaparib and the QUADRA clinical trial for niraparib (Oza et al., 2017; Moore et al., 2019). Rucaparib was approved for advanced *BRCA1/2* mutated ovarian carcinomas following multiple chemotherapy treatments, while niraparib was approved for homologous recombination deficiency (HRD)-positive gynecological cancers (Franzese et al., 2019; Kim and Nam, 2022). Following the initial approvals, Phase III clinical trials continued to demonstrate the efficacy of PARP inhibitors in gynecological cancers.

The SOLO2 clinical trial was a Phase III, double-blinded, randomized, placebo-controlled study that evaluated the use of olaparib as maintenance therapy in platinum-sensitive, relapsed ovarian cancer patients with a *BRCA1/2* mutation who had received at least two lines of previous chemotherapy (Pujade-Lauraine et al., 2017). After at least 4 cycles of platinum-based treatment, 196 of 295 patients were randomly assigned to receive either 300 mg of olaparib twice daily in tablet form or a placebo. In the trial group, 67% of these patients had confirmed germline *BRCA1* mutations, while 30% had *BRCA2* mutations and the remaining patients had missing information regarding their mutation status. The olaparib group’s median progression-free survival (PFS) was 19.1 months, compared to 5.5 months in the control group. The trial also found that olaparib had manageable toxicities and no detrimental effects on the quality of life in these patients. This trial eventually led to the approval of olaparib for *BRCA1/2*-mutated ovarian cancer patients with at least two lines of previous chemotherapy (Franzese et al., 2019).

The SOLO1 trial was also a Phase III, double-blinded, randomized study that evaluated the use of olaparib as maintenance therapy for patients with newly diagnosed high-grade serous or endometrioid ovarian cancer, primary peritoneal cancer, or fallopian tube cancer (or a combination thereof) with *BRCA1/2* mutations who had responded to first time platinum-based chemotherapy (Moore et al., 2018; DiSilvestro et al., 2022). In the initial trial, of the 391 enrolled patients, 260 were randomly assigned to receive 300 mg of olaparib twice daily, while the remaining 131 received a placebo (Moore et al., 2018). Median PFS for patients receiving a placebo was 13.8 months; meanwhile for those receiving olaparib was 56.0 months. This trial eventually led to the approval of olaparib for this indication. A 7-year follow-up of these patients further support the use of olaparib to slow down the remission for this indication (DiSilvestro et al., 2022).

The ENGOT-OV16/NOVA Phase III clinical trial was a randomized, double-blinded, placebo-controlled study of niraparib as maintenance in platinum-sensitive ovarian cancer patients with either germline *BRCA* (*gBRCA*) mutation or a tumor with high-grade serous histology who have responded to their most recent chemotherapy containing a platinum agent (Mirza et al., 2016). For this review, we will focus on the *gBRCA* cohort, which contained 203 patients, 138 of whom were in the niraparib group and were given 300 mg of niraparib daily instead of a placebo. Of the treatment group, 61.6% had BRCA1 mutations, 37.0% had BRCA2 mutations, and 6.5% had either BRCA1 or BRCA2 rearrangements, or both. In the *gBRCA* cohort, the median PFS was 21.0 months compared to 5.5 months in the placebo group. The most common severe adverse effects of niraparib were thrombocytopenia, anemia, and neutropenia, controlled with dose modification (Mirza et al., 2016; Berek et al., 2018). The results of this trial played a crucial role in the FDA approval of niraparib for the maintenance therapy of adult patients with recurrent epithelial ovarian, fallopian tube, or primary peritoneal cancer who are in complete or partial remission following platinum-based chemotherapy (Franzese et al., 2019).

The ARIEL3 Phase III clinical trial was a randomized, double-blind, placebo-controlled trial that evaluated the efficacy of rucaparib as a maintenance therapy for women with platinum-sensitive ovarian cancer who had achieved a complete or partial response to second-line or later platinum-based chemotherapy (Coleman et al., 2017). This trial had multiple cohorts, but for the purpose of this review, we will focus on the *BRCA1/2*-mutant cohort. Out of 196 patients randomized in this group, 130 were given a daily regimen of rucaparib 600 mg twice daily, while the remainder received a placebo. The treatment group of the *gBRCA*1/2 cohort comprised 61.5% *BRCA1* mutants and 38.0% *BRCA2* mutants. The median progression-free survival was significantly longer in the rucaparib group at 16.6 months compared to 5.4 months in the placebo group. This trial was pivotal in granting rucaparib FDA approval in 2018 for maintenance therapy of recurrent epithelial ovarian, fallopian tube, or primary peritoneal cancers in complete or partial remission to platinum-based chemotherapies.

The ARIEL4 Phase III clinical trial was a randomized, controlled, open-label study that sought to evaluate the efficacy of rucaparib as an alternative treatment option to chemotherapy for patients with relapsed, *BRCA1/2*-mutated ovarian cancers (Kristeleit et al., 2022). Among the multiple cohorts included in this trial, the present review focuses on the efficacy population, which excluded patients with *BRCA*1/2 reversion mutations. Of the 325 patients in the efficacy group, 220 received oral rucaparib 600 mg twice daily in 28-day cycles, regardless of platinum sensitivity status. The treatment group consisted of 79% *BRCA1* mutants and 21% *BRCA2* mutants. The median progression-free survival was significantly longer in the rucaparib group at 7.4 months compared to 5.4 months in the placebo group. Overall, the results of the ARIEL4 study support the use of rucaparib as a viable alternative to chemotherapy for patients with relapsed, *BRCA1/2*-mutated ovarian carcinoma.

### 2.2 Breast cancer

Breast cancer is the second most common cancer in the world (Ferlay et al., 2019), which has a wide range of risk factors that include but are not limited to family history, age, and environmental factors (Collaborative Group on Hormonal Factors in Breast, 2001; Cortesi et al., 2021). Approximately 5–10% of patients diagnosed with breast cancer have an inherited loss of function in one or both *BRCA1* and *BRCA2* genes (Couch et al., 2014). FDA has approved two PARP inhibitors, olaparib and talazoparib, for therapeutic use in breast cancers with germline and somatic *BRCA1/2* mutations.

The OlympiAD clinical trial was a Phase III, open-labeled, randomized, multicenter, international study that evaluated the use of olaparib as monotherapy for the treatment of HER2-negative metastatic breast cancer with germline *BRCA1/2* mutations (Robson et al., 2017; Robson et al., 2019). Of the 302 enrolled patients, 205 were randomly assigned to receive 300 mg of olaparib twice daily, while the remaining 97 received standard therapy (capecitabine, eribulin, or vinorelbine). In the trial group, *BRCA1* germline mutations were present in 57.1% of the patients, *BRCA2* mutations were present in 41.0% of the patients, and 2% of the patients carried mutations in both genes. The trial included participants who received less than two prior chemotherapy regimens for metastatic disease. Participants were selected because they had received neoadjuvant, adjuvant, or treatment for metastatic disease with an anthracycline (unless contraindicated) and a taxane. Patients were excluded if the disease relapsed within 12 months of adjuvant platinum. In the trial, olaparib outperformed standard therapy regarding median PFS time and toxicity. PFS was 7.0 months in the trial group and 4.2 months in the standard therapy. The risk of disease progression or death was 42% lower with olaparib monotherapy than with standard therapy. Olaparib also had fewer grade 3 or higher adverse events.

The Olympi**A** double-blinded, randomized Phase III trial was performed to determine if olaparib could reduce the rate of recurrence of cancers in individuals with *BRCA1/2* mutations (Tutt et al., 2021). The 1836 individuals were randomly assigned to receive olaparib maintenance for a year or a placebo. In these patients, *BRCA1* germline mutations were present in 71.3% of the patients, *BRCA2* mutations were present in 28.3% of the patients, and 0.2% of patients carried mutations in both genes. Olaparib outperformed the standard therapy and was associated with significantly longer invasive disease-free survival (IDFS) and distant disease-free survival (DDFS) than the standard therapy. Specifically, 3-year DDFS was 7.1% greater in the olaparib group compared to the placebo group, while 3-year IDFS was 8.8% greater in the olaparib group. In addition, fewer deaths were reported in the olaparib group. This trial determined that olaparib could be used as maintenance therapy for early breast cancer patients who are HER2-negative and have *BRCA1/2* germline mutations. Follow-ups from the OlympiA clinical trial demonstrated a statistically significant improvement in overall survival with olaparib treatment, with an absolute improvement in 4-year overall survival of 3.4%, with 89.8% of patients in the olaparib group surviving compared to 86.4% in the placebo group. The participants maintained improvements in IDFS and DDFS (Geyer et al., 2022). This trial led to the approval of olaparib as a maintenance therapy for *BRCA1/2* mutated breast cancers.

The Phase III EMBRACA trial was a randomized, open-label trial that led to the FDA approval of talazoparib for breast cancer patients (Litton et al., 2018; Litton et al., 2020). Talazoparib monotherapy was used in this trial to treat HER2-negative metastatic breast cancers with germline *BRCA1/2* mutation. In this trial, 287 out of 431 participants were given 1 mg talazoparib once daily, and the rest of the patients were given standard therapy. In the talazoparib group, *BRCA1* germline mutations were present in 46.3% of the patients and *BRCA2* mutations were present in 53.7% of the patients. Participants were selected if they had received no more than three previous cytotoxic regimens for advanced breast cancer. They received prior treatment with a taxane, an anthracycline, or both unless this treatment was contraindicated. Patients were excluded if they had objective disease progression while receiving platinum chemotherapy for advanced breast cancer. The median PFS among patients in the talazoparib group was approximately 2.8 months longer than in the standard therapy group, with a median PFS of 7.0 months in the talazoparib group and 4.2 months in the standard therapy group.

### 2.3 Prostate cancer

Prostate cancer is the most common cancer and the second leading cause of cancer death in men in the United States. The incidence of prostate cancer varies widely among different populations. In general, the rates of prostate cancer are the highest in North America and Europe and lowest in Asia and Africa (Pernar et al., 2018). The exact reason for these differences is not well understood but is likely due to a combination of genetic, environmental, and lifestyle factors. Of the inherited prostate cancers, approximately 5.3% contain *BRCA2* and 0.9% contain *BRCA1* mutations making these subsets of prostate cancer ideal targets for PARP inhibitor therapy (Messina et al., 2020). In May 2020, the FDA granted accelerated approval for rucaparib based on the TRITON2 Phase II trial for the treatment of adult patients with metastatic castrate-resistant prostate cancer (mCRPC) associated with a deleterious BRCA mutation (germline and/or somatic) who have received androgen receptor-directed therapy and a taxane (Abida et al., 2020; Anscher et al., 2021). Olaparib also received approval based on the results of the Phase III PROfound trial (de Bono et al., 2020).

After the Phase III PROfound trial, olaparib monotherapy was approved for use in patients with mCRPC who have progressed after receiving prior treatment with enzalutamide or abiraterone and have detrimental germline or somatic homologous recombination repair (HRR) gene mutations (de Bono et al., 2020). This trial examined multiple HRR genes and looked closely at *BRCA1*, *BRCA2,* and *ATM* mutations in cohort A of this study. In cohort A, 162 patients were randomly assigned to receive olaparib and 83 to the control treatment. *BRCA1* germline mutations were present in 5% of the patients, *BRCA2* mutations were present in 49% of the patients, and 37% had *ATM* mutations. The remaining patients had a combination of the three mutations. For patients in cohort A, the median duration of follow-up *via* imaging-based PFS was 7.5 months in the olaparib group and 5.4 months in the control group. The study found that the median overall survival for cohort A was 18.5 months in the group receiving olaparib treatment, while it was 15.1 months in the control group. Exploratory analyses of this trial suggested that patients with *BRCA1* or *BRCA2* alterations derived the most benefit.

### 2.4 Pancreatic cancer

Pancreatic cancer is a relatively rare but aggressive form of cancer, and it is often difficult to diagnose in its early stages. The outlook for pancreatic cancer depends on the cancer stage at the time of diagnosis, but it is generally considered a very serious and difficult-to-treat cancer with a 5-year survival of approximately 9%. *BRCA1/2* mutations have been identified in familial pancreatic cancers, with the majority being germline *BRCA2* gene mutations, which occur in approximately 5–17% of patients with familial pancreatic cancer (Rawla et al., 2019). As a result, this subset of pancreatic cancers is a prime target for treatment with PARP inhibitors.

Currently, the only FDA-approved PARP inhibitor to treat pancreatic cancers is olaparib. The approval is based on the POLO Phase III clinical trial that was randomized, double-blind, and placebo controlled (Golan et al., 2019). In this study, 92 patients out of 154 participants were given olaparib (300 mg, twice daily), while the remainder were given a placebo. In these patients, *BRCA1* germline mutations were present in 32% of the patients, *BRCA2* mutations were present in 67% of the patients, and 1% of the patients carried mutations in both genes. After 2 years, olaparib treatment prevented tumor development in 22.1% of patients with chemotherapy-responsive BRCA1/2-mutated malignancies. In comparison, only 9.6% of individuals receiving the placebo had no tumor development. In addition, the median PFS was 7.4 and 3.8 months after treatments with olaparib and the control medication, respectively. Overall, the POLO trial showed that maintenance olaparib provided significant benefits to patients with a *BRCA1/2* mutation and metastatic pancreatic cancer that had not progressed during platinum-based chemotherapy (Golan et al., 2019; Kindler et al., 2022).

### 2.5 Companion testing for clinical trial eligibility

PARP inhibitors have shown promising results in treating cancers that have genetic defects in homologous recombination repair (HRR), particularly those related to BRCA mutations. To determine which patients are most likely to respond to these therapies, various genetic tests have been developed to identify variant, deletion, or duplication mutations in the BRCA1 and BRCA2 genes (Toland et al., 2018). Several of the trials discussed in the previous section rely on the Homologous Recombination Deficiency (HRD) tests, which assess the degree of HRD in cancer cells by the presence of genomic scars (Ngoi and Tan, 2021). During phase III clinical trials, patient selection was based on the presence of BRCA mutations, and different testing methods were used based on availability. The FoundationOne CDx next-generation sequencing test and the BRACAnalysis CDx Germline Companion Diagnostic Test were used in some trials, and both have been FDA-approved as companion tests to match patients with specific PARP inhibitor drugs. An in-depth review of these techniques to detect BRCA-mutations was recently described by Concolino, P. & Capoluongo (Concolino and Capoluongo, 2019).

The BRACAnalysis CDx was first approved by the FDA in conjunction with the PARP inhibitor Olaparib, specifically for the selection of BRCA mutations in ovarian cancer patients. This diagnostic tool is designed to detect and classify variants in the germline BRCA1 and BRCA2 genes, including single nucleotide variants (SNVs) and small insertions or deletions, through PCR and Sanger sequencing. The BRACAnalysis CDx also includes an extensive rearrangement test, known as BART CDx, which detects major genomic rearrangements using multiplex PCR. The complete sequence analysis of the BRCA1 and BRCA2 genes is carried out using the BRACAnalysis CDx by evaluating approximately 5400 and 10,200 base pairs, respectively. This test examines the coding and non-coding regions of the BRCA1 and BRCA2 genes (Gunderson and Moore, 2015). The second FDA-approved companion test for determining eligibility for PARP inhibitors is the FoundationOne CDx next-generation sequencing test. This test utilizes the Next Generation Sequencing (NGS)-based comprehensive genomic profiling technology to analyze 324 cancer genes in solid tumors, including BRCA. The FoundationOne CDx reports known and likely pathogenic short variants, copy number alterations, select rearrangements, as well as complex biomarkers such as tumor mutational burden, microsatellite instability, and genomic loss of heterozygosity in ovarian cancer (Milbury et al., 2022). These tests can be used for a multitude of cancers, making them a crucial tool. A comprehensive list of FDA-approved companion tests and their approved use can be seen at the following link: https://www.fda.gov/medical-devices/in-vitro-diagnostics/list-cleared-or-approved-companion-diagnostic-devices-in-vitro-and-imaging-tools. Given the complex nature of repair defects and the multitude of proteins involved, it is not surprising that there is still no established gold standard for HRD-deficiency detection (Ngoi and Tan, 2021). Consequently, there is a pressing need to develop more reliable diagnostic technology to test for the underlying genomic signatures associated with successful use of PARP inhibition.

## 3 Molecular mechanism behind the clinical efficacy of PARP inhibitors in treating the *BRCA1/2* mutant cancers

Faithfully replicating and repairing its genome is vital for the fitness and health of a mammalian cell. Replisomes frequently encounter a variety of impediments throughout the genome. Transient pausing, stalling, and collapsing of replication forks trigger a series of signaling transduction events, which are commonly referred to as the replication stress response (Zeman and Cimprich, 2014; Berti and Vindigni, 2016; Saxena and Zou, 2022). The well-recognized endogenous DNA replication impediments include unrepaired DNA lesions, mis-incorporated ribonucleotides, repetitive DNA sequences that are prone to form various secondary DNA structures (e.g., G-quadruplex or G4), a collision of a replication fork with the transcription machinery (i.e., transcription-replication conflict, or TRC), R-loops that are formed between nascent transcribed RNAs and their adjacent displaced single-stranded DNA (ssDNA), DNA-protein complexes, tightly packed genomic regions (e.g., heterochromatin), and others (Zeman and Cimprich, 2014). Due to the prevalence of various types of replication impediments, replisomes are much more prone to pause, stall, or even collapse at certain difficult-to-replicate (DTR) genomic regions, including centromeres, common fragile sites (CFS), rDNA loci, and telomeres (Table 3).

**TABLE 3:** Shared features of the four difficult-to-replicate (DTR) regions in human genome.

	Telomere	Common fragile site	Centromere	rDNA loci
**Chromosome locations**	All chromosomes; 2 each; ∼10–15 kb	Cell type dependent; 10–20 most sensitive and unstable sites in the APH-treated cells; up to ∼5 Mb	All chromosomes; 1 each; up to 1 Mb	1q, 13p, 14p, 15p, 21p, 22p; *50* kb *to > 6* Mb
**Repetitive sequences**	(TTAGGG)_n_	AT-rich microsatellites	Alpha satellites (171 bp AT-rich)_n_	rDNA gene repeats, repetitive enhancer elements, satellite repeats
**Secondary DNA structures**	Yes (G4s)	Yes (hairpins)	Yes (hairpins)	unknown
**Stable DNA-protein complex**	Yes (Shelterin)	Yes (CTCF)	Yes (CENP-B)	Yes (TTF-I & Timeless)
**R loops**	Yes (TERRA R-loops)	Yes	Yes (α-satellite R-loops)	Yes
**Heterochromatin**	Yes	Yes	Yes	Yes
**Late replicating**	Yes	Yes	Yes	Yes
**Replication origin**	Poor	Poor	Poor	unknown
**Ultra fine bridge**	Yes	Yes	Yes	Yes
**HDR/BIR/MMEJ**	Yes	Yes	Yes	Yes
**BRCA1/2**	Yes	Yes	Yes	Yes
**PARP1**	Yes	Yes	Yes	Yes

Many cancers constantly experience more heightened replication stress compared to normal cells. The reasons include: (1) dysregulated coordination between cell cycle progression and DNA replication due to the loss of tumor suppressors, such as p53 and Rb, and the overexpression of many oncogenes, such as Myc and Ras; (2) mutations in genes encoding the DNA damage response (DDR) and DNA repair proteins, such as *BRCA1* and *BRCA2*. The heightened replication stress in many cancers has been hypothesized to be their Achilles’ heel and can potentially be used as a target for cancer treatment (Cybulla and Vindigni, 2022; da Costa et al., 2022).

BRCA1 and BRCA2 play an essential role in repairing the double-stranded DNA breaks (DSBs) *via* the homologous recombination (HR) (Roy et al., 2012). In addition, we and others have shown that they also function in suppressing the replication stress by facilitating the repair and re-start of the stalled replication fork (Pathania et al., 2011; Schlacher et al., 2011; Schlacher et al., 2012; Tian et al., 2013). PARP1 belongs to a family of 17 enzymes that catalyze the ADP-ribosylation reaction and plays a versatile role in various DNA metabolism (Gani et al., 2015; Huang and Kraus, 2022). Because all the PARPi competitively target the active site, in theory, they can inhibit multiple PARPs (Rudolph et al., 2022). However, using PARP1 knockout cells, Hopkins and colleagues showed that PARP1 is the primary target in causing cytotoxicity in *BRCA1/2* mutant cancers (Hopkins et al., 2019). Intriguingly, in addition to inhibiting its catalytic activity, PARPi can also trap PARP1 on DNA (Satoh and Lindahl, 1992; Kedar et al., 2012; Murai et al., 2012; Rudolph et al., 2022), suggesting that the trapped PARP1-PARPi complex physically impedes the progression of replication machinery and induces replication stress, thus indirectly contributing to their cytotoxic effects. The degree of PARP1 or PARP2 trapping has also been implicated in the reason for the different potencies of the various clinical PARPi (Murai et al., 2012).

In this section, we discuss the hypothesis that when treated with PARPi, the BRCA1/2 deficient tumors experience intolerable replication stress at multiple DTR loci, which then leads to cell death.

### 3.1 The role of BRCA1, BRCA2, and PARP1 in suppressing the replication stress at telomeres

One of the well characterized DTR loci that frequently poses challenges to the replisome is the telomere. Mammalian telomeres consist of tandem repetitive DNA sequences, (TTAGGG)_n_, located at the end of each linear chromosome. The length of human telomeres varies from 10 to 15 kb (Blasco, 2005). The enzyme telomerase catalyzes the *de novo* synthesis of TTAGGG at the shortened telomeres. Telomerase is a large ribonucleoprotein (RNP) complex and it elongates telomeres by copying its RNA component, hTR, with its intrinsic reverse transcriptase activity. 85–90% of cancers manifest robust telomerase activity (TEL+) (Kim et al., 1994; Shay and Bacchetti, 1997). However, in 10–15% of cancers, the telomerase activity is undetectable yet these cancer cells still can elongate their telomeres. These cancers adopt the so-called Alternative Lengthening of Telomere (ALT) pathway to maintain their telomeres (Bryan et al., 1995; Bryan et al., 1997).

**FIGURE 1:**
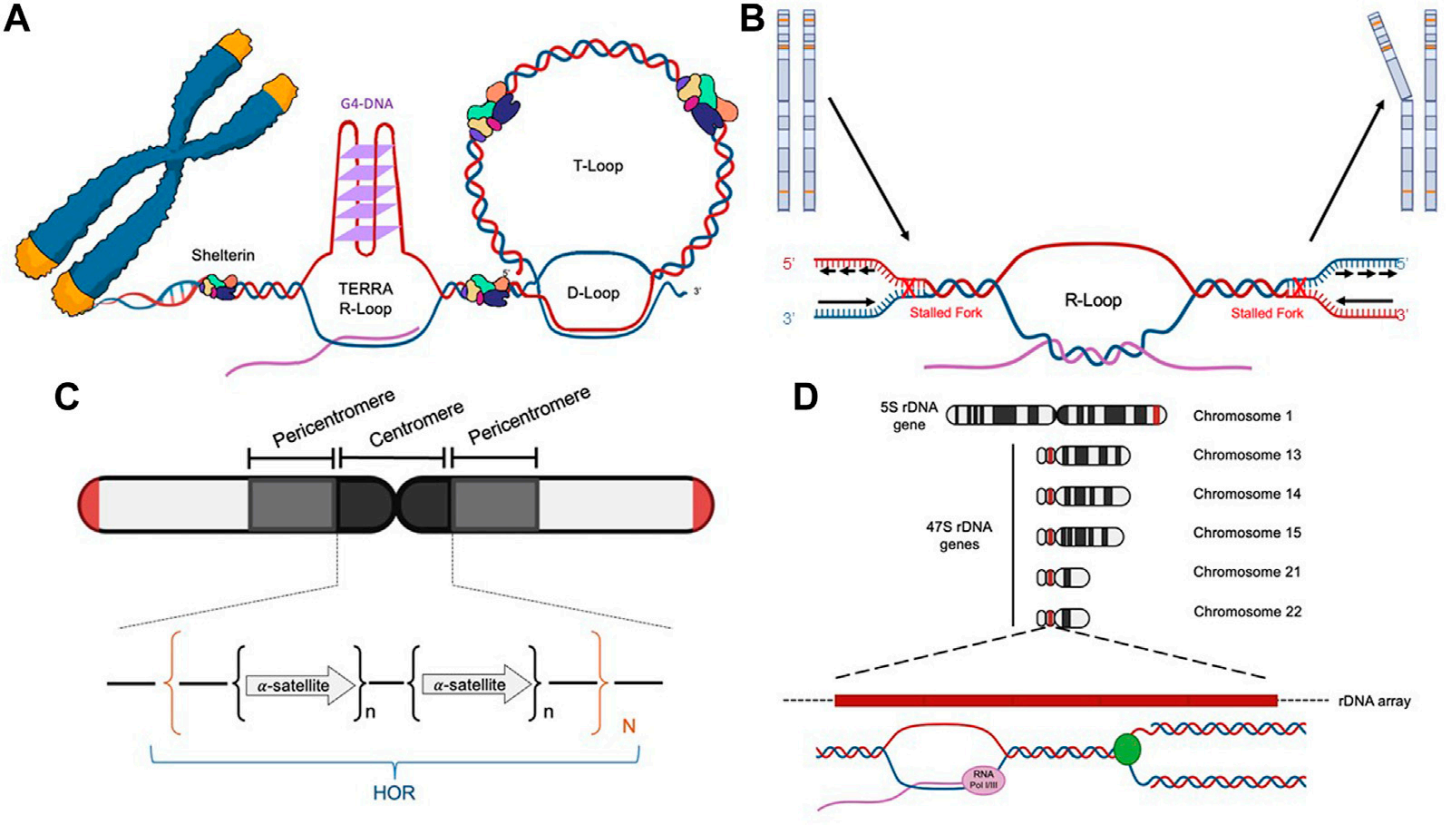
Diagrams of the four difficult-to-replicate (DTR) loci in human genomes: telomeres **(A)**, common fragile sites **(B)**, centromeres **(C)**, and rDNA loci **(D)**.

The unique sequence and structural features of human telomeres render them especially challenging to replisomes (Figure 1A and Table 3). (1) One of the strands of telomere is rich in guanines (G) (thus called the G-rich strand) and is prone to form G4s (Biffi et al., 2013; Lam et al., 2013). (2) The subtelomeric/telomeric long non-coding RNA, TERRA, has been shown to form R-loops with telomeric DNA (Azzalin et al., 2007; Schoeftner and Blasco, 2008). (3) It was shown by Drosopoulos and colleagues that DNA replication can be initiated within telomeres; however, the large majority of DNA replication near the end of mammalian chromosomes is initiated from subtelomeres (Drosopoulos et al., 2012), suggesting that most telomeres lack DNA replication origins. Furthermore, Sfeir and colleagues showed that treatment with a low dosage of aphidicolin, a reversible DNA polymerase inhibitor, dramatically increases the incidence of fragile (broken) telomeres (Sfeir et al., 2009). These observations indicate that telomeres may be a special type of CFS. Furthermore, telomeres in the ALT-positive (ALT+) cells are more heterogeneous in length and some of them can be quite long (Bryan et al., 1995; Bryan et al., 1997). Therefore, ALT+ telomeres may be even more prone to express the fragility. (4) Heterochromatins are enriched at subtelomeres and telomeres (Blasco, 2007). (5) In order to protect the end of linear chromosomes, there are various high order DNA-DNA and DNA-protein structures at telomeres. For example, the telomeric G-rich strand overhang folds back, invades the internal double-stranded regions of telomeres, and forms the telomeric-loop (T-loop) as well as the displacement-loop (D-loop) (Griffith et al., 1999; Doksani et al., 2013). The T-loop and D-loop are further stabilized by the Shelterin complex and other proteins (de Lange, 2005; Pfeiffer and Lingner, 2013). These various high-order DNA-DNA and DNA-protein structures also slow down the replisome. Taken together, telomeres pose much greater challenges to the replisome than other regions of the human genome. Indeed, replication stress and spontaneous DNA damages are frequently detected at telomeres, especially in ALT+ cells (Cesare et al., 2009; Sfeir et al., 2009; Suram et al., 2012).

BRCA1 associates with telomeres in both TEL+ (Ballal et al., 2009) and ALT+ cells (Wu et al., 2003). Intriguingly, BRCA1 is recruited in higher amounts to ALT telomeres that experience heightened replication stress (Conomos et al., 2014; Pan et al., 2017). Mechanistically, our lab has shown that in coordination with BLM and FANCM, BRCA1 suppresses the TERRA R-loop accumulation-induced replication stress at the ALT telomeres and is required for the survival of ALT+ cancer cells (Pan et al., 2017). Moreover, Vohhodina and colleagues showed that BRCA1 directly binds TERRA RNA and also suppresses its expression in the ALT+ cancer cells (Vohhodina et al., 2021).

BRCA2 has also been shown to be implicated in DNA metabolism at telomeres. For example, BRCA2 facilities telomere synthesis/replication in TEL+ HeLa cells (Min et al., 2012). Moreover, it was also shown to facilitate the recruitment of Rad51, a key factor in HR, to telomeres in a unique HeLa derived clone, HeLa 1.2.11, which has super long telomeres (van Steensel et al., 1998; Badie et al., 2010). In mouse embryonic fibroblast (MEF) cells, deficiency of BRCA2 induces telomere fragility and telomere shortening (Badie et al., 2010; Min et al., 2012). Interestingly, Kwon and colleagues showed that BRCA2 deficiency facilitates the activation of the ALT pathway in telomerase-null mouse cells (Kwon et al., 2019). Consistent with these findings, we showed that BRCA2 is required for the formation of C-circles, an important ALT biomarker, in ALT+ cells that experience severe replication stress at their telomeres (Pan et al., 2019). Most intriguingly, Lee and colleagues demonstrated recently that BRCA2 directly binds the telomeric G4s *in vitro*, suggesting that it may be involved in disrupting the G4 accumulation, thereby preventing replication fork stalling at telomeres (Lee et al., 2022).

PARP1 plays a crucial role in maintaining the integrity of telomeres (Muoio et al., 2022). PARP1 can also be found at telomeres in both unstressed and IR- and H_2_O_2_-treated HeLa 1.2.11 (Gomez et al., 2006). In addition, PARP1 also associates with and PARylates TRF2 *in vivo*, a key component of the Shelterin complex. Interestingly, when Shelterin is removed, the repair of telomeric DNA damage requires the PARP1-dependent alternative non-homologous end joining pathway (alt-NHEJ) when the canonical NHEJ (cNHEJ) pathway is not available (Sfeir and de Lange, 2012). Furthermore, PARP1-dependent alt-NHEJ also contribute to the repair of telomere-internal DSBs induced by the endonuclease, Fok I (Doksani and de Lange, 2016). Finally, a recent study showed that PARP1 modulates the biogenesis and activity of telomerase through two RNA-binding proteins, DKC1 and GAR1(Savelyev et al., 2021).

Taken together, we propose that in BRCA1/2 deficient tumors, telomeres are more prone to unresolved replication stress and subsequent DNA damage. When these tumors are treated with PARPi, the combination of abrogated PARP1 catalytic activity-mediated events and the dramatic increase in trapped PARP1-PARPi DNA complexes leads to further blockage of replisome progression. This exacerbates the replication stress at telomeres and eventually kills the BRCA1/2 deficient cells.

### 3.2 The role of BRCA1, BRCA2, and PARP1 in suppressing the replication stress at common fragile sites

Common fragile sites (CFSs) are identified as genomic regions that are prone to manifest ssDNA gaps in the metaphase spread assay when treated with low concentrations of aphidicolin, which slows but does not completely inhibit DNA replication (Glover et al., 1984; Hecht and Glover, 1984). The prominent features of CFSs include (Figure 1B and Table 3) (Glover et al., 2017): (1) enriching in deoxyadenosine and thymidine (AT-rich), which are prone to form hairpin DNA secondary structures; (2) enriching in long genes up to ∼5Mb; the transcription of the long genes could lead to formation of R-loops; (3) replicating late; and (4) lacking of replication origins.

In both pathogenic *BRCA1* mutant cancer cells and BRCA1 siRNA depleted cancer cells, Arlt and colleagues showed that two CFSs, FRA3B and FRA16D, manifested elevated expression of the fragility compared to wild-type *BRCA1* complemented or control siRNA transfected cells (Arlt et al., 2004). Consistent with these findings, Turner and colleagues reported that the loss of expression of Fhit, encoded by the long gene *FHIT* at the common fragile site, FRA3B, was more frequent in the BRCA1 mutant breast cancers than the sporadic breast cancers (Turner et al., 2002). They further showed that the BRCA1 deficient mouse cells also manifest more breaks and gaps at multiple CFSs. Similarly, Berthorsson and colleagues observed higher levels of genetic aberrations and allelic imbalance at FRA3B in the *BRCA2* mutated hereditary breast cancers than in the sporadic breast cancers (Bergthorsson et al., 1998).

Using the metaphase spread assay, Vernole and colleagues did not observe any increased DNA breaks and gaps in the mismatch repair (MMR)-deficient colorectal cancers when PAPR1 was inactivated either with PARPi or siRNA when compared to the MMR-proficient colorectal cancers (Vernole et al., 2011). Similarly, using the spectral karyotyping technique, Lavoie and colleagues did not observe elevated expression of fragility in PARP1-null mouse cells either (Lavoie et al., 2005). However, using the recently developed Strand-seq technology, which can map genome-wide sister chromatid exchange (SCE) *via* single-cell sequencing, Heijink and colleagues showed that PARPi-induced SCEs frequently take place at many CFSs (Heijink et al., 2022). Notably, they demonstrated that these SCE events are independent of BRCA1 and BRCA2, suggesting that PARP1 likely acts in a parallel pathway facilitating the repair of replication blockage at the CFSs.

Collectively, strong evidence indicates that BRCA1, BRCA2, and PARP1 are all implicated in preventing replication stress and facilitating the repair/re-start of stalled replication forks at the CFSs. PARP1 likely functions in a parallel pathway of BRCA1 and BRCA2 in these molecular processes. Therefore, inhibition of PARP1 would further exacerbate the replication stress at the CFSs in the BRCA1/2 deficient tumors.

### 3.3 The role of BRCA1, BRCA2, and PARP1 in suppressing the replication stress at centromeres

Centromeres are essential chromosome regions where kinetochores are assembled during mitosis and meiosis to ensure the equal partition of chromosomes (Barra and Fachinetti, 2018). However, because of the highly enriched various repetitive sequences, the identification of the exact sequences in the centromeric regions has been challenging for the short-read based sequencing technologies, such as Sanger sequencing and Next-Generation Sequencing (NGS). With the recently developed long-read sequencing technologies, such as Oxford Nanopore Technology (ONT), more precise sequence identification of the centromeric regions has been allowed. Two excellent review articles discussed how the exciting new telomere-to-telomere (T2T) human genome data would have a profound impact on the centromere research (Miga, 2020; Miga and Alexandrov, 2021). In general, there are a few prominent sequence features of the centromeric regions in humans (Figure 1C and Table 3): (1) enriching of various DNA repeats, including the alpha satellite repeats and long interspersed nuclear elements (LINEs); (2) spanning over several megabases long; (3) enriching of heterochromatin; (4) enriching of secondary DNA structures.

BRCA1 can be found at centrosomes throughout the cell cycle (Pageau and Lawrence, 2006; Zhu et al., 2011; Di Paolo et al., 2014). Intriguingly, Racca and colleagues showed that the formation of R-loops at the centromeric alpha satellite repeats further facilitates the association of BRCA1 with centromeres (Racca et al., 2021). Centromeric BRCA1 helps to prevent the accumulation of the centromeric alpha satellite R-loops formation (Racca et al., 2021). Consistent with these studies, Yilamz and colleagues showed that in G1 cells, DSBs at centromeres increase the R-loop formation, which then leads to the recruitment of BRCA1 and Rad51 to the centromeres and suppression of centromere instability through the activation of HR. Most importantly, Zhu and colleagues showed that BRCA1 deficiency induces over-active transcription of the alpha satellite repeats and alters the heterochromatin at the centromere, leading to increased DNA damage at centromere (Zhu et al., 2011).

PARP1 can also be found at active centromeres (Earle et al., 2000; Saxena et al., 2002b). For example, in mouse embryonic stem cells, robust interactions can be detected between PARP1 and CENP-A, and between PARP1 and CENP-B, two proteins that play a crucial role in maintaining the heterochromatin status at centromeres (Saxena et al., 2002a). Intriguingly, Gemble and colleagues showed that in the cytidine deaminase deficient cells, an important enzyme that is involved in the pyrimidine salvage pathway, excessive dCTPs can also inhibit PARP1, leading to DNA replication defects and formation of the ultrafine anaphase bridges (UFBs) *via* centromeres and common fragile sites (Gemble et al., 2015). The formation of UFBs are indicative of incomplete DNA replication or entangled HR intermediates (Bizard and Hickson, 2018). These data demonstrated a crucial role of PARP1 in the DNA replication at centromeres and the common fragile sites, likely through remodeling the heterochromatin status (Dantzer and Santoro, 2013).

### 3.4 The role of BRCA1, BRCA2, and PARP1 in suppressing the replication stress at rDNA loci

Ribosomes are cellular machinery where all the cellular proteins are translated. Ribosomes are ribonucleoprotein complex (RNPs) that are made up of many proteins and RNAs, which are called ribosomal proteins and ribosomal RNA (rRNA) respectively. In humans, there are four different rRNAs: 5S, 18S, 5.8S, and 28S, which are transcribed by RNA polymerase I and III. The 5S rRNA (∼150 nucleotides) is transcribed from a cluster of repeated 2.2 kb long genes on 1q42 (Sorensen et al., 1991). The other three rRNAs (18S, 5.8S, and 28S) are generated through post-transcriptional processing of a 45S precursor RNA transcribed from clusters of repeated 43 kb long genes on 13p12, 14p12, 15p12, 21p12, and 22p12 (Henderson et al., 1972). The genomic regions that encode the rRNA are collectively called the rDNA loci (Figure 1D and Table 3), which are the most actively transcribed genomic regions in proliferating human cells (Lane and Fan, 2015). The increased transcriptional activity at the rDNA loci greatly increases the likelihood of collision between the two cellular machineries: the RNA polymerase complexes responsible for transcription and the DNA polymerase complexes responsible for DNA replication. This collision is known as the transcription-replication conflict (TRC).

Using chromatin immunoprecipitation (ChIP) assay, Johnston and colleagues demonstrated that BRCA1 associates with the rDNA loci in multiple breast cancer cell lines (Johnston et al., 2016). Additionally, they also showed that BRCA1 interacts with RNA polymerase I and regulates the transcription of rRNAs. Intriguingly, a recent study by Chang and colleagues showed that the C-terminus domain of BRCA1, i.e., the BRCT domain, facilitates the hybridization of rRNA and the antisense-rRNA thus preventing the formation of R-loops between rRNA and rDNA, and avoiding DNA damage at the rDNA loci (Chang et al., 2022).

Also using the ChIP assay, Guetg and colleagues showed that PARP1 also associates with the rDNA loci (Guetg et al., 2012). Interestingly, they also found that PARP1 binds TIP5, a key component of NoRC and facilitates the re-establishment of heterochromatin in the rDNA loci after their DNA replication.

## 4 Potential challenges and mechanisms associated with PARPi resistance in the clinic

As discussed in Section 2 and summarized in Table 2, there are clear clinical benefits in using the PARPi to treat *BRCA1/2* mutated ovarian, breast, prostate, and pancreatic cancers, however, they are not a panacea for all the aforementioned malignancies. One of the most significant challenges is the rapid development of drug resistance to these inhibitors.

Clinically, there are at least two types of PARPi resistance mechanisms: HDR-dependent and HDR-independent. The HDR-dependent mechanisms include: (1) reversion mutants in the *BRCA1/2* genes that partially or fully restore their HDR function (Goodall et al., 2017; Quigley et al., 2017; Pettitt et al., 2020); (2) inactivation of certain subunits of the shieldin complex (e.g., 53BP1), which normally functions to shield the DSB from being resected, leading to increased DNA end resection activity in the *BRCA1* mutated tumors (Cruz et al., 2018; Dev et al., 2018). The HDR-independent mechanisms include: (1) mutations in PARP1 genes that compromised the catalytic inhibition or PARP trapping activity of the PARPi (Pettitt et al., 2018); (2) dysregulation of the turnover of parylated proteins, for example through the inactivation of PARG (Gogola et al., 2018); (3) inactivation of additional DDR proteins, for example SLFN11 (Cruz et al., 2018). Therefore, exploring additional synthetic lethal interactions with BRCA1/2 may overcome the PARPi resistance (Patel et al., 2021).

## 5 PARPi and immunotherapy

Despite the promising advances in basic science and clinical trials that have helped us understand the mechanisms surrounding PARP inhibitors, further research is needed to safely translate these findings into the best possible patient outcomes. To further improve the efficacy of PARPi in the clinic, many early clinical trials combining PARPi with immunotherapy agents, such as immune checkpoint blockades (ICBs) are actively undergoing. Here, we briefly discuss some of the ongoing clinical trials investigating combination approaches in BRCA1/2 deficient cancers. Early success in these trials underscores the importance of promoting anti-tumor immune responses, which are primed in DNA damage settings. Further discussion of the potential molecular mechanisms and immune pathways can be found in additional reviews (Concannon et al., 2023).

In the TOPACIO/KEYNOTE-162 Phase II trial, investigators tested niraparib in combination with pembrolizumab in patients with triple-negative breast cancer or ovarian cancer (Vinayak et al., 2019). Intriguingly, endpoint analysis of the breast cancer cohort in this study determined that there was more pronounced activity in *BRCA1/2* mutant patients, as determined by ORR rates of 47% compared to 11% and PFS of 8.3 months *versus* 2.1 months, respectively. However, this pattern of results was not observed in the ovarian cancer cohort of the same trial. Though the combination treatment did show promising antitumor activity, the trial did not meet the overall endpoint ORR criteria and there was no significant difference in patients with or without BRCA mutations (Konstantinopoulos et al., 2019).

In the JAVELIN BRCA/ATM Phase IIb trial, the combination of avelumab and talazoparib was tested in patients with *BRCA1/2* mutations (159 out of 200 patients, 79.5%) or *ATM* mutations (41 out of the 200 patients, 20.5%) (Schram et al., 2022). Although this study did not achieve the pre-specified goal of an OR rate of 40%, the OR rate in the *BRCA1/2* mutation cohort is much higher (26.4%) than that in the *ATM* mutation cohort (4.9%). A separate JAVELIN PARP Phase I/II trial tested the combination of avelumab and talazoparib in patients with various advanced solid tumors including the *BRCA1/2* mutant ovarian cancers (Yap et al., 2022). Most intriguingly, the *BRCA1/2* mutant ovarian cancers respond much more frequently than other types of cancers. Overall, the promising JAVELIN trial results warrant further investigation in future randomized clinical trials.

The MEDIOLA open-label, Phase I/II trial tested the safety and activity of olaparib plus durvalumab in patients with germline and non-germline mutated *BRCA1/2* metastatic breast cancers (Domchek et al., 2020). While the overall data showed no clear associations between clinical outcomes and factors such as HRD, genomic instability status, and PD-L1 status the study included a triple combination of olaparib and durvalumab plus the antiangiogenic agent, bevacizumab. This triple treatment cohort had the highest disease control rate (DCR) and the longest median survival. Importantly, white blood cell counts must be continually monitored during combination therapies as a potential for lymphopenia can cause patients to be susceptible to certain infections.

Taken together, these recent Phase II trials suggest that the *BRCA1/2* mutated cancers may benefit from the combination approaches of PARP inhibitors and immunotherapy agents. Therefore, further clinical trials are warranted to confirm the benefits of the combined therapies in patients with *BRCA1/2* mutations. Additionally, the reason for the proclivities of certain cancers to be more susceptible to the combination treatments should be investigated, as was the case in the TOPACIO trial. Results from the first phase III clinical trial, ATHENA-COMBO, are expected to be published in the near future, which examine the outcomes of rucaparib plus nivolumab. Furthermore, additional combinations of PARPi and other agents that have not been mentioned in this section (such as checkpoint inhibitors) are being studied and could prove valuable in the design of complementary DNA damage response inhibition strategies.

## 6 Conclusion

A better understanding of the molecular mechanism of how the PARPi selectively targets the BRCA1/2 deficient cells will be informative to develop novel targeted therapies. Here, we briefly summarized the data supporting the hypothesis that BRCA1, BRCA2 and PARP1 play an important role in suppressing the replication stress at multiple DTR genomic regions. We propose that in the BRCA1/2 deficient tumors, there is already heightened replication stress at the DTR loci. When treated with PARPi, the replication stress is further exacerbated and becomes intolerable, eventually leading to cell death. On the other hand, predicating the sensitivity to PARPi and pinpointing the precise mechanism of the response can be challenging in the clinic. For example, in a recent publication, Hill and colleagues established a panel of patient-derived ovarian cancer organoids and attempted to predict how well they respond to various DNA repair inhibitors, including the olaparib, using a variety of molecular, cellular and genomic biomarkers (Hill et al., 2018). Among the 10 organoids derived from five patients with germline *BRCA1/2 mutation*, only one was sensitive to olaparib. Intriguingly, among the 9 olaparib-resistant organoids, 6 manifested the HDR signature but were also positive for the Rad51 foci, while 5 manifested unstable stalled replication fork. This new study underscores the importance in the continuing effort to search for the most reliable biomarker(s) for PARPi response as well as the molecular mechanism of their action in order to maximize the clinical benefits for cancer patients while minimize the healthcare cost.

Finally, the combined treatment of PARPi and immunotherapy agents may further improve the efficacy of PARPi and renders a more hopeful perspective for the *BRCA1/2* mutant cancer patients.
